# Episiotomy characteristics and risks for obstetric anal sphincter injuries: a case-control study

**DOI:** 10.1111/j.1471-0528.2012.03293.x

**Published:** 2012-05

**Authors:** M Stedenfeldt, J Pirhonen, E Blix, T Wilsgaard, B Vonen, P Øian

**Affiliations:** a The Norwegian Continence and Pelvic Floor Centre, University Hospital of North NorwayTromsø, Norway; b Department of Clinical Medicine, Faculty of Health Science, University of TromsøTromsø, Norway; c Department of Clinical Research, University Hospital of North NorwayTromsø, Norway; d Department of Health and Caring Sciences, Faculty of Health Science, University of TromsøTromsø, Norway; e Department of Community Medicine, Faculty of Health Science, University of TromsøTromsø, Norway; f Nordland Hospital BodøBodø, Norway; g Department of Obstetrics and Gynaecology, University Hospital of North Norway TromsøNorway

**Keywords:** Episiotomy, episiotomy technique, obstetric anal sphincter injuries, vaginal birth

## Abstract

**Objectives:**

To investigate the association between the geometrical properties of episiotomy and obstetric anal sphincter injuries (OASIS) because episiotomies angled at 40–60° are associated with fewer OASIS than episiotomies with more acute angles.

**Design:**

Case–control study.

**Setting:**

University Hospital of North Norway, Tromsø and Nordland Hospital, Bodø, Norway.

**Sample:**

Seventy-four women who had one vaginal birth and episiotomy. Cases (*n* = 37) have sustained OASIS at birth, while controls (*n* = 37) had not. The groups were matched for instrumental delivery.

**Methods:**

Two groups of women with history of only one vaginal birth were compared. Episiotomy scar was identified and photographed and relevant measures were taken. Data were analysed using conditional logistic analysis.

**Main outcome measures:**

Mean episiotomy angle, length, depth, incision point.

**Results:**

The risk of sustaining OASIS decreased by 70% (odds ratio [OR] 0.30; 95% CI 0.14–0.66) for each 5.5-mm increase in episiotomy depth, decreased by 56% (OR 0.44; 95% CI 0.23–0.86) for each 4.5-mm increase in the distance from the midline to the incision point of the episiotomy, and decreased by 75% (OR 0.25; 95% CI 0.10–0.61) for each 5.5-mm increase in episiotomy length. Lastly, there was no difference in mean angle between groups but there was a “U-shaped” association between angle and OASIS (OR 2.09; 95% CI 1.02–4.28) with an increased risk (OR 9.00; 95% CI 1.1–71.0) of OASIS when the angle was either smaller than 15°or >60°.

**Conclusion:**

The present study showed that scarred episiotomies with depth > 16 mm, length > 17 mm, incision point > 9 mm lateral of midpoint and angle range 30–60° are significantly associated with less risk of OASIS. Shrinkage of tissue must be considered.

## Introduction

Obstetric anal sphincter injuries (OASIS) are the most common causes of anal incontinence in women. OASIS is a severe perineal tear during vaginal delivery, causing partial or total rupture of the anal sphincter.^1^ Approximately 30–50% of women affected will suffer from anal incontinence after primary repair.^2^, ^3^ This can be both physically and mentally devastating.^4^ Several obstetrical factors associated with increased risk of sustaining OASIS have been identified,^4^ and although debated, episiotomy is often described as one.^4^, ^5^

In most Western countries, episiotomy is recommended only on indication.^6^ This is also recommended by the guidelines in Norway and the current episiotomy rates at our birth units are between 12 and 15%. The purpose of episiotomy is to expedite delivery in the case of fetal distress, increase the area of passage in cases of instrumental deliveries or shoulder dystocia, or to minimise the risk of OASIS.^7^

There are several variations of episiotomies, and three types are commonly described. (1) Medial episiotomy is performed as an incision dividing the perineal tissue in the midline down toward the anal canal.^5^ (2) Mediolateral episiotomy has its incision point at the midline raising an angle between 40 and 60° to the left or right of the anal canal.^7^–^10^ (3) Lateral episiotomy has its incision point to the left or right of the midline, at either 4–5 or 7–8 o’clock and the cut is angled 40–60° from the midline.^11^

There is strong evidence against midline episiotomy, showing a clear association with increased risk of OASIS.^5^, ^12^, ^13^ Consensus is lacking regarding the role of mediolateral episiotomy.^5^ Recent studies indicate that mediolateral episiotomy is protective against OASIS^10^ both during operative vaginal deliveries^14^ and for primiparous women.^15^ However, Andrews et al.^8^ performed a prospective study showing that mediolateral episiotomy is a strong risk factor for perineal trauma. Two large cohort studies indicate that mediolateral episiotomy is protective in first vaginal delivery, whereas for the second or higher vaginal delivery it is not.^15^, ^16^

Questions about the specific technique as well as about the common definition of mediolateral episiotomy have been raised.^7^, ^11^, ^17^ Kalis et al.^11^ revealed how definitions of mediolateral episiotomy used in European hospitals had a low degree of agreement and a vast range of individual interpretation. Other studies have shown how health professionals perform mediolateral episiotomies differently, causing some episiotomies to lie closer to the midline than intended.^7^, ^9^, ^17^ This discrepancy in definition and technique highlights the question whether inaccurate conclusions might have been drawn, and if it might be misleading to compare reports.^5^, ^17^

However, studies evaluating the episiotomy technique revealed that the angle is significantly associated with OASIS and that such injuries seem to occur more often when the angle of the mediolateral episiotomy is <40°.^9^, ^17^–^20^

We decided to investigate episiotomy characteristics defined from angle, length, depth and incision point. The aim of the study was to determine the association between these episiotomy characteristics and OASIS.

## Methods

### Design and study population

The study was carried out at the University Hospital of North Norway and Nordland Hospital, Norway. We performed a matched case–control study using the electronic patient journal system Partus® (CSAM Health AS, Lysaker, Norway), which identified eligible participants. Obstetric information for all births was obtained by a search from 2004 to 2011. Women were initially included in the study if they had one vaginal delivery only and an associated episiotomy.

This group was further subdivided using Sultan’s classification for OASIS.^21^

Women with clinically identified third-degree or fourth-degree tears at birth, graded as 3a, 3b, 3c or 4, were classified as cases whereas the controls were women with no OASIS. Women in the cases and controls groups where matched for ventouse/forceps because of the strong association between OASIS and instrumental delivery).^8^, ^16^, ^22^ A power calculation using results from Andrews et al.^8^ was undertaken for episiotomy angle, with an anticipated difference of 11° between groups, with a standard deviation of 13°, giving a significance level of 5% and power of 90%. The sample size required 37 women in each group.

Fifty-three women with OASIS and 75 matched control women were contacted and asked to participate by letter and phone-call. A total of five women were excluded because of language or pregnancy and 49 (16 OASIS cases and 33 matched controls) women declined to participate. Seventy-four eligible women were willing to participate in the study. The included women signed a written informed consent and were called in for a physical examination.

### Techniques

**Figure 1:**
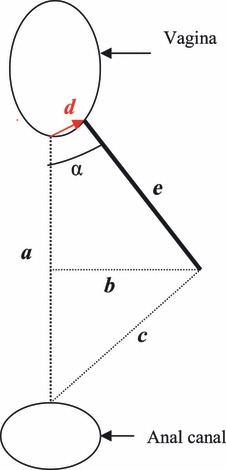
Adapted from Andrews et al.^17^which illustrates the measurements taken. *a* = line drawn from the posterior fourchette to the outer edge of the anal epithelium; *b* = episiotomy depth, a line from the caudal end of the episiotomy bisecting line ‘*a*’ perpendicularly; *c* = the shortest distance from the caudal end of the episiotomy to the anterior outer edge of the anal epithelium; *d* = line from the posterior fourchette to the point of episiotomy incision and *e* = episiotomy length.

This study attempted to measure the lines and angle given between the fixed points of the posterior fourchette, the episiotomy and the most anterior point of the anal epithelium, named distances *a*, *b*, *c*, *d*, *e* and angle *α* (Figure 1). The distances were measured in millimetres, and the angle refers to *x*/360 of a full circle. The distance *a* (midline) was defined as the length between the posterior fourchette to the anal canal, distance *b*: the depth from the caudal end of the episiotomy intercepting line ‘*a*’ perpendicularly, *c*: the shortest distance from the caudal point of the episiotomy to the anal canal, *d*: the distance from the posterior fourchette to the incision point of the episiotomy. Episiotomy length, *e*, was defined as the length from the outer rim of the labia, fully exposed, but not excessively stretched and angle (*α*), the angle between *a* (midline) and the episiotomy.

**Figure 2:**
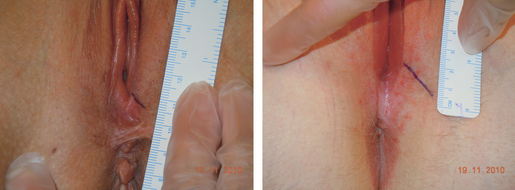
Digital picture visualising two episiotomies with different geometric properties. Left picture shows an episiotomy associated with an increased risk of sustaining OASIS, whereas the episiotomy in the right picture has the angle, length, depth and incision point associated with less risk.

At the physical examination, the vaginal introitus/perineum was assessed for the episiotomy scar, and a picture was taken. All women were in a semi-lithotomy position with legs resting in knee holders. A Nikon Coolpix S8000 camera was used. The camera was fixed on a tripod and the procedure instructed the camera lens to be levelled horizontally, with a 40-cm distance from the introitus. Aim of focus was the vaginal opening, and the anal and vaginal openings were included in the photograph in addition to the episiotomy (Figure 2). For measurement reference a surgical ruler (CODMAN) was included in the photograph, positioned vertical to the episiotomy.

The episiotomy scar was marked with a surgical pen (CODMAN) before the photograph was taken. To annotate the photographs and draw all lines between the fixed points as in Figure 1 an Adobe Photoshop version CS5 (Adobe Systems Inc, San Jose, CA, USA) Exclusive was used. All the photographs were taken by the first author (MS) and four photographs were taken of each woman. Two experienced obstetricians (PØ, JP) blinded for cases and controls did the selection of the photographs, one for each case and control. All the relevant lines and angles for further measures were drawn twice into two identical sets of photographs. The lines were drawn by an externally hired computer drafter once together with the investigator (MS) and once together with a blinded experienced obstetrician (PØ). The drafter measured the double set of lines on separate occasions. This study was approved by the Regional Ethics Committee North Norway (163/2008).

### Statistics

Agreement between the two readings was evaluated using a Bland–Altman plot, intraclass correlation coefficient (ICC) and coefficient of variation (CV). An ICC < 0.90 indicates very good agreement, CV < 15% is recognised as sufficient.^23^–^25^ Data were analysed using spss version 18 (SPSS Inc, Chicago, IL, USA).

A conditional logistic regression model was used to assess differences between cases and controls and to calculate odds ratio (OR) for OASIS. The OR was estimated per SD increase in continuous variable. Pairwise combinations of episiotomy characteristics were simultaneously included in the regression models to identify associations adjusted for other episiotomy characteristics. A significance level of 0.05 was set throughout. Associations were adjusted for birthweight.

To look at associations between OASIS and angle ranges, and very narrow/very wide angles we transformed the continuous variable ‘angle’ into categories (0–15, 16–30, 31–45, 46–60, >60°) and a dichotomous variable with intervals: 15–60 and <15 or >60°. Both crude and adjusted OR were estimated. Further, to capture a possible ‘U-shaped’ association between the continuous variable angle and OASIS, a linear and quadratic term was tested. For sensitivity analysis extreme values of birthweight were identified and excluded. All statistical analyses were then executed again without the outliers.

## Results

Descriptive characteristics in the cases and controls are reported in Table 1. Women sustaining OASIS gave birth to children with significantly higher mean birthweight compared with women with no such injuries (3764 versus 3377 g, *P* = 0.009). Mean head circumference was also significantly higher in the case group compared with the controls (36.9 versus 35.9 cm, *P* = 0.036).

**Table 1:** Descriptive characteristics of women in case and control groups

	Case group (*n* = 37) OASIS	Control group (*n* = 37) No OASIS	*P* value**

Mothers age (years) at delivery*	30 (6.3) 26–36	29 (6.4) 24–35	0.46
Birth weight (g)*	3764 (662) 3259–4254	3376 (472) 3183–3712	0.01
Head circumference (cm)*	36 (1.6) 35–37	35 (1.8) 34–36	0.04
Time from birth to assessment (years)*	2.9 (1.8) 1.0–4.5	2.2 (1.1) 1.3–2.9	0.06
**OASIS**			
3a	11 (30)	–	
3b	13 (35)	–	
3c	8 (21)	–	
4	5 (14)	–	

* The values are mean (standard deviation) and interquartile range (IQR) or *n* (%).

** *P* values from conditional regression.

Interobserver reliability was based on replicated drawings and measures of all pictures. ICC was 0.88 (95% CI 0.80–0.92), and CV was 15.4% for distance *a*; ICC was 0.99 (95% CI 0.99–0.99) and CV was 6.7% for distance *b*; ICC was 0.98 (95% CI 0.94–0.98) and CV was 9.7% for distance *c*; ICC was 0.92 (95% CI 0.78–0.90) and CV was 29.9% for distance *d*; ICC was 0.98 (95% CI 0.96–0.99) and CV was 8.5% for *e*; and ICC was 0.99 (95% CI 0.99–0.99) and CV was 7.9% for episiotomy angle. For all measures, a Bland–Altman plot was created. Combining ICC, CV and Bland–Altman plot distances *b*, *c*, *d*, *e* and angle gave a high level of agreement whereas distance *a* showed only a moderate level of agreement.

Univariable conditional logistic regression analyses revealed significant differences between cases and controls (Table 2). Mean distance *b* was smaller in the case group (11 mm) than in the control group (16 mm). This was also the case for mean distance *d* (6 versus 9 mm*)* as well as mean episiotomy length *e* (13 versus 17 mm). The mean angle did not differ between the two groups. However, a significant ‘U-shaped’ association between angle and OASIS was captured (OR 2.09, 95% CI 1.02–4.28) and when the angle was transformed to a dichotomous variable such as angle narrower than 15° or wider than 60° versus angle range 15–60°, there was a significant difference between the groups. Only six (16%) women without OASIS had an episiotomy angle <15 or >60°, whereas 14 (38%) women sustaining OASIS showed an episiotomy angle <15 or >60°.

**Table 2:** Odds ratio of sustaining obstetric anal sphincter rupture based on the characteristics of the episiotomy*

Characteristics	Cases (*n* = 37) OASIS**	Controls (*n* = 37) No OASIS**	OR (95% CI)	OR adjusted for birthweight (95% CI)	*P* value

Distance of perineal body (*a*) (mm)***	27 (8) 21–31	24 (6) 20–26	1.36 (0.85–2.19)	1.20 (0.70–2.07)	0.51
Episiotomy depth (*b*) (mm)***	11 (5) 8–13	16 (6) 13–20	0.30 (0.14–0.66)	0.27 (0.10–0.72)	0.01
Distance from caudal end of episiotomy to anal canal (*c*) (mm)***	26 (9) 21–34	25 (9) 19–32	1.06 (0.65–1.72)	1.04 (0.58–1.86)	0.90
Distance from midpoint of posterior fourchette to incision point of episiotomy (*d*) (mm)***	6 (4) 4–9	9 (5) 6–12	0.44 (0.23–0.86)	0.43 (0.20–0.95)	0.04
Length of episiotomy (*e*) (mm)***	13 (5) 8–16	17 (6) 12–21	0.25 (0.10–0.61)	0.23 (0.08–0.66)	0.01
Angle of episiotomy***	43 (29) 25–55	43 (19) 26–51	1.00 (0.58–1.76)	1.05 (0.54–2.04)	0.90
Angle of episiotomy <15 or >60°****	14 (38%)	6 (12%)	9.00 (1.1–71.0)	9.19 (1.07–78.50)	0.04

* Conditional logistic regression; women in the case and control groups are matched for instrumental delivery.

** The values are mean (standard deviation) and interquartile range.

*** Odds ratio per standard deviation of the independent variable.

**** Odds ratio of OASIS for value very narrow/wide angle versus angles ranged from 15 to 60°.

The odds ratio estimates show that there is a 70% reduced risk (OR 0.30, 95% CI 0.14–0.66) of sustaining an anal obstetric sphincter rupture for each 5.5-mm increase in depth of the episiotomy. In addition, by increasing the distance from the posterior fourchette to the incision point of the episiotomy by 4.5 mm the risk for an obstetric anal injury decreases by 56% (OR 0.44, 95% CI 0.23–0.86). Also, there is a 75% reduced risk (OR 0.25, 95% CI 0.10–0.61) of obstetric anal injuries for each 5.5-mm increase in the length of the episiotomy, and there is an increased risk of sustaining such an injury when the angle is either <15 or >60° (OR 9.0, 95% CI 1.1–71.0). Angle categorised as 0–15° (OR 10.03, 95% CI 0.68–147.64), 16–30° (OR 1.12, 95% CI 0.22–5.76), 31–45° (reference), 46–60° (OR 0.75, 95% CI 0.16–3.56) and >60° (OR 8.88, 95% CI 0.59–133.83) did not reach significance.

The results remained the same after adjusting for birthweight. Excluding two extreme values of birthweight (5254 and 1922 g) from the calculation did not alter the findings.

In a separate set of multivariable models we included pairwise combinations of the significant continuous variables *b*, *d* and *e*. In the model with *d* and *b*, *d* was not significantly associated with OASIS (*P* = 0.41), whereas depth *b* was (*P* = 0.01). In the model with *d* and *e*, *d* was not significant (*P* = 0.19), whereas length, *e* was (*P* = 0.01) and in the model with depth *b* and length *e* there was a non-significant trend for both measures towards association with OASIS (*b*: *P* = 0.07; length: *P* = 0.06).

## Discussion

This case–control study demonstrated that point of incision, length and depth as well as angle are all parameters associated with anal sphincter injury. Incisions very close to the posterior fourchette, short episiotomies, angles smaller than 15° or larger than 60° and short depth are factors that increase the risk of third-degree and fourth-degree perineal tears. In other words the protective effects seem to depend on the whole construct of the geometric figure created by the episiotomy and the fixed point of the vagina and the anal canal.

Further, depth and episiotomy length were the most significant characteristics associated with less risk of OASIS when compared with incision point. Our findings might indicate that to unload the perineum sufficiently the episiotomy must obtain a certain length and depth.

The length (*a*) between the posterior fourchette and the anal opening was not significantly different between the groups, neither was distance *c*, the length from the caudal end of the episiotomy to the anal canal. The lack of significant difference between the groups for distance *a*, suggests that the primary repair after the injury has managed to maintain the perineal body for the case group.

The significant association between OASIS and episiotomy length, point of incision, depth and angle persisted regardless of the adjustments made for birthweight or when outliers were excluded. This corresponds with earlier findings.^9^

Only a few reports have controlled or described the episiotomy technique and reported comparable results.^7^, ^9^, ^17^–^20^ Tincello et al.^7^ was the first to question the technique of mediolateral episiotomy and to raise the issue of the degree of force relief upon the perineum related to the angle of the episiotomy. The investigators theorised that an episiotomy angle of 30° or less would function as a midline incision thus not unload the perineum sufficiently. The authors also proposed that an extremely wide angled episiotomy might cause the same lack of relief of pressure upon the perineum. With a pictorial questionnaire the authors demonstrated that the assumption that episiotomy technique was the same for all caregivers was not valid. They also found that physicians exerted both longer and more angled episiotomies than midwives.

Further, in an observational study by Andrews et al.^8^ episiotomy angle was measured immediately after repair. It was demonstrated that episiotomies angled more acutely (26 versus 37°) were associated with a significant increase in the risk of OASIS. A case-control study with a total of 100 primiparas had similar findings.^9^ The cases were 54 women sustaining a third degree tear, and 46 women with no injury constituted the control group. The authors found that the mean angle was significantly smaller in the case (30°) group than in the control group (38°).

Our results also show a variation in practice. Although being aware of perineal changes and shrinkage after birth we were still surprised by the considerable variation in episiotomy techniques. Figure 2 demonstrates some of the variations: short episiotomy close to the midline, compared with an episiotomy with measures associated with the less risk of sustaining OASIS.

Also, supporting the theory of Tincello et al.,^7^ we confirmed that both very small angles (<15°) and extremely large angles (>60°) were associated with OASIS. We found a ‘U-shaped’ association between angle and OASIS, suggesting that a scarred episiotomy angle ranging from 30 to 60° imposes the least risk of OASIS. Considering shrinkage and changes of perineal tissue after birth, our results support the findings of other clinical studies.^8^, ^9^

Poor correlation between the angle of episiotomy at the time of the cut and the scarred episiotomy angle measured at postnatal visit is reported in two studies. According to Kalis et al.^18^ there is an episiotomy shrinkage of 12° (mean) after 6 months. A similar shrinkage was observed by van Dillen et al.^20^ The authors inspected episiotomies in 25 women immediately after birth and compared them with measurements made at a postnatal control. The mean angle of the episiotomy was 38.6 ± 7.8° compared with 31.2 ± 11.5°, respectively. According to Andrews et al.,^8^ a 26° angle was significantly associated with obstetric anal injury compare with a 37° angle, measured immediately after delivery. Considering these two results (26 − 12°), a scarred episiotomy angle of 15° would resemble an episiotomy angle at birth associated with increased risk of obstetric anal sphincter injuries. On the other hand, a 60° angle will probably resemble an episiotomy angle of >70° at the time of birth.

To our knowledge, this is the first study to investigate if incision point of the episiotomy away from midline is associated with OASIS. In Finland, a lateral episiotomy has been the method of choice for several decades. It has been speculated that this tradition has been associated with low OASIS rates. However, this assumption has been made through a large cohort study.^16^ A significant reduction in OASIS frequency was seen after an intervention programme conducted at five birth units in Norway.^26^, ^27^ The intervention consisted of a clinical training programme aimed at the birth team with four primary teaching aims. One was to perform lateral episiotomy instead of mediolateral, and on indication only.^26^

Results from our study indicate that women with an episiotomy for which the incision point was lateral to the midline had less risk of OASIS than those women with an episiotomy with the incision point close to the midline (Figure 2). The benefit of the incision point being away from the midline could be a consequence of directing pressure away from the midline already at the incision point.

Unlike our study, Andrews et al.^8^ did not find a significant association between the episiotomy length and OASIS. They suggested that a longer episiotomy with a large angle would cause a longer distance to the anal canal, which could decrease the risk of OASIS. This study suggests that distance from the end of the episiotomy to the anal canal (distance *c*) is not a characteristic that is associated with OASIS. Depth (distance *b*) on the other hand, was the characteristic with the strongest association with OASIS. When considering the geometric structure of the midline, vaginal opening, episiotomy incision point and episiotomy length, the characteristic depth *b* is perhaps the most explanatory because it is a function of lengths *d* and *e* and angle *α*.

Episiotomy technique is a modifiable procedure, and it is therefore important to make note of the parameters referred to in this study. Taking these into consideration and accommodating them into practice can potentially lead to a reduction in OASIS. Forty-eight percent of the episiotomies in the case group and 16% in the control group did not have characteristics such as depth > 16 mm, length > 17 mm, incision point > 9 mm lateral of midpoint and angle range 30–60°, which were significantly associated with less risk of OASIS. This highlights the need for supervised and standardised hands-on training.

This study has limitations. With 37 women in each group it is a small study. The results must therefore be interpreted with caution. As the measures studied are collected some time after birth, we do not know the true measures of the episiotomy at birth. At the time of birth the perineal distension and oedema caused by the crowning of the head, cause the perineum to be larger than at the time of repair and postnatal control. Therefore, the measures in this study will necessarily be smaller than at the time of performing the episiotomy. One hundred and 28 women were asked to participate in the study, 16 (13%) women in the case group and 33 (26%) in the control group did not want to be included. More control women were unwilling to participate than women in the case group. This might have influenced our results, which need to be confirmed in future studies and preferably in a randomised controlled trial.

## Conclusion

This case–control study supports other studies of episiotomy technique,^7^– ^9^, ^19^, ^20^ indicating that narrow-angled episiotomies increase the risk of OASIS. In addition, our findings suggest that measures such as point of incision, episiotomy length and depth might reduce the risk for OASIS. The episiotomy’s depth and length are of particular importance. This is a small study, and technique-specific research with a larger sample is needed to further investigate the effect of geometric measures on OASIS.
